# Identifying Aboriginal-specific AUDIT-C and AUDIT-3 cutoff scores for at-risk, high-risk, and likely dependent drinkers using measures of agreement with the 10-item Alcohol Use Disorders Identification Test

**DOI:** 10.1186/1940-0640-9-17

**Published:** 2014-09-01

**Authors:** Bianca Calabria, Anton Clifford, Anthony P Shakeshaft, Katherine M Conigrave, Lynette Simpson, Donna Bliss, Julaine Allan

**Affiliations:** 1 National Drug and Alcohol Research Centre, University of New South Wales, Sydney, NSW, Australia; 2 School of Population Health, University of Queensland, Brisbane, QLD, Australia; 3 Drug Health Services, Royal Prince Alfred Hospital, Missenden Road, Camperdown, NSW, Australia; 4 Sydney Medical School, University of Sydney, Sydney, NSW, Australia; 5 Drug Health Services, South Western Sydney Local Health District, Croydon Health Centre, Croydon, NSW, Australia; 6 Yoorana Gunya Family Healing Centre Aboriginal Corporation, Forbes, NSW, Australia; 7 The Lyndon Community, Orange, NSW, Australia

**Keywords:** AUDIT, AUDIT-C, AUDIT-3, Alcohol, Measures, Aboriginal

## Abstract

**Background:**

The Alcohol Use Disorders Identification Test (AUDIT) is a 10-item alcohol screener that has been recommended for use in Aboriginal primary health care settings. The time it takes respondents to complete AUDIT, however, has proven to be a barrier to its routine delivery. Two shorter versions, AUDIT-C and AUDIT-3, have been used as screening instruments in primary health care. This paper aims to identify the AUDIT-C and AUDIT-3 cutoff scores that most closely identify individuals classified as being at-risk drinkers, high-risk drinkers, or likely alcohol dependent by the 10-item AUDIT.

**Methods:**

Two cross-sectional surveys were conducted from June 2009 to May 2010 and from July 2010 to June 2011. Aboriginal Australian participants (N = 156) were recruited through an Aboriginal Community Controlled Health Service, and a community-based drug and alcohol treatment agency in rural New South Wales (NSW), and through community-based Aboriginal groups in Sydney NSW. Sensitivity, specificity, and positive and negative predictive values of each score on the AUDIT-C and AUDIT-3 were calculated, relative to cutoff scores on the 10-item AUDIT for at-risk, high-risk, and likely dependent drinkers. Receiver operating characteristic (ROC) curve analyses were conducted to measure the detection characteristics of AUDIT-C and AUDIT-3 for the three categories of risk.

**Results:**

The areas under the receiver operating characteristic (AUROC) curves were high for drinkers classified as being at-risk, high-risk, and likely dependent.

**Conclusions:**

Recommended cutoff scores for Aboriginal Australians are as follows: at-risk drinkers AUDIT-C ≥ 5, AUDIT-3 ≥ 1; high-risk drinkers AUDIT-C ≥ 6, AUDIT-3 ≥ 2; and likely dependent drinkers AUDIT-C ≥ 9, AUDIT-3 ≥ 3. Adequate sensitivity and specificity were achieved for recommended cutoff scores. AUROC curves were above 0.90.

## Background

Problem drinkers consume alcohol at levels that increase their risk of causing physical and psychological harm to themselves, their family, and their community [1]. Problem drinkers’ alcohol consumption patterns can also be referred to as problematic alcohol use. Although Aboriginal Australians are more likely to abstain from drinking alcohol than other Australians, a greater proportion of Aboriginal Australians who drink alcohol do so at levels that increase their risk of alcohol-related harm [2,3]. Screening Aboriginal people to assess their level of alcohol consumption is recognized as an important initial step for determining their risk of alcohol-related harm and the need for alcohol intervention [4-6]. Alcohol screening can also be effective for engaging Aboriginal patients in discussions about their drinking [7] and can result in reduced alcohol consumption, independent of intervention [8,9].

The Alcohol Use Disorders Identification Test (AUDIT) was developed by the World Health Organization as a cross-cultural screening instrument for problematic alcohol use [10,11]. AUDIT has 10 items comprising 3 domains: recent alcohol use; alcohol dependence symptoms; and alcohol-related problems [11]. Cutoff scores aim to identify nondrinkers, low-risk drinkers, at-risk drinkers, high-risk drinkers, and likely dependent drinkers [11-13]. AUDIT has high internal consistency across diverse samples and settings (median alpha = 0.83) and demonstrated validity for the English-language version [14]. Although AUDIT has not been formally validated in the Aboriginal Australian population, the Alcohol Treatment Guidelines for Indigenous Australians recommend using AUDIT to screen for alcohol use problems among Aboriginal Australians [5]. The Guideline’s recommended classification scores are 0–7 for nondrinkers or low-risk drinkers, 8–12 for at-risk drinkers, and 13+ for high-risk drinkers. A key limitation of AUDIT for routine screening in Aboriginal-specific [7,15] and mainstream [16] health care settings has been the time it takes respondents to complete all 10 items. Two shorter versions of AUDIT, AUDIT-C (comprising the first three items of AUDIT) and AUDIT-3 (the third item of AUDIT), have been shown to perform well in identifying problem drinking when compared with a ‘gold standard’ measure of problem drinking, for example, with DSM-IV alcohol dependence criteria [17], in non-Indigenous-specific health care settings [14].

Despite evidence from qualitative studies that shorter versions of AUDIT are more feasible to deliver in Aboriginal-specific primary health care settings [7,15,18], and that these shorter versions are being used to measure Aboriginal Australians’ drinking in general practice settings [18], no published studies have identified cutoff scores specifically for Aboriginal Australians. This paper aims to identify AUDIT-C and AUDIT-3 cutoff scores for at-risk, high-risk, and likely dependent drinkers to give health care providers a better understanding of the strengths and limitations of AUDIT-C and AUDIT-3 for identifying problem drinkers in Aboriginal people in primary health care.

## Methods

### Ethics

Ethics approval for the study was granted by: the Human Research Ethics Committee (HREC), University of New South Wales (NSW), Sydney; South West Area Health Service HRECs, Sydney; and the Aboriginal Health and Medical Research Council Ethics Committee, NSW. The study was also either formally approved by the board of the participating Aboriginal Community Controlled Health Services (ACCHSs) or had a representative of the ACCHS on its steering committee. All participants were provided with study and consent information.

### Setting and participants

A convenience sample of Australian Aboriginal participants (age 18 years or older) was recruited through a NSW rural ACCHS and a rural community-based drug and alcohol treatment agency from July 2010 to June 2011, as part of a study investigating the acceptability of an evidence-based cognitive-behavioral alcohol intervention to Aboriginal people [19]. These participants were recruited through existing community-based groups run by the ACCHS (58% of the sample) or clients of the drug and alcohol treatment agency who were seeking treatment (11% of the sample). Participants were also recruited through existing Aboriginal community-based groups in metropolitan Sydney from June 2009 to May 2010, as part of a pilot study of community education and brief intervention [20]. The groups were approached by researchers and offered an interactive education session about alcohol and pre-education screening (30% of the sample). Participants recruited in rural NSW were reimbursed $A40 to cover their out-of-pocket expenses for involvement in the study. Reimbursement was not available for participants in the Sydney-based sample.

### Questionnaires

A pen-and-paper version of the 10-item AUDIT previously modified for and proven to be acceptable to Aboriginal Australians (Table 1) [20] was self-completed by participants, with literacy support available from researchers and, in some cases, health care providers if required. Surveys were typically completed in a public space (waiting room or group room); however, participants were not required to write their name on survey forms. In the rural sample, participants completed AUDIT as part of a larger survey. In the urban sample, AUDIT was completed by participants before interactive education session results were provided to individual participants at the end of the session.

**Table 1:** Alcohol Use Disorders Identification Test (AUDIT) - adapted wording for Aboriginal Australians

	**Adapted Aboriginal-specific AUDIT items **[20]	**Original AUDIT item**	**Response**	**Score**
1.	How often do you drink?	How often do you have a drink containing alcohol?	Never	0
			Monthly or less	1
			2–4 times a month	2
			2–3 times a week	3
			4 or more times a week	4
2.	When you have a drink, how many do you usually have in one day?	How many standard drinks containing alcohol do you have on a typical day when drinking?	1 or 2	0
			3 or 4	1
			5 or 6	2
			7–9	3
			10 or more	4
3.	How often do you have six or more drinks on one day?	How often do you have six or more drinks on one occasion?	Never	0
			Monthly or less	1
			Monthly	2
			Weekly	3
			Daily or almost daily	4
4.	In the last year, how often have you found you weren’t able to stop drinking once you started?	During the past year, how often have you found that you were not able to stop drinking once you had started?	Never	0
			Monthly or less	1
			Monthly	2
			Weekly	3
			Daily or almost daily	4
5.	In the last year, how often has drinking got in the way of doing what you need to do?	During the past year, how often have you failed to do what was normally expected of you because of drinking?	Never	0
			Monthly or less	1
			Monthly	2
			Weekly	3
			Daily or almost daily	4
6.	In the last year, how often have you needed a drink in the morning to get yourself going?	During the past year, how often have you needed a drink in the morning to get yourself going after a heavy drinking session?	Never	0
			Monthly or less	1
			Monthly	2
			Weekly	3
			Daily or almost daily	4
7.	In the last year, how often have you felt bad about your drinking?	During the past year, how often have you had a feeling of guilt or remorse after drinking?	Never	0
			Monthly or less	1
			Monthly	2
			Weekly	3
			Daily or almost daily	4
8.	In the last year, how often have you had a memory lapse or blackout because of your drinking?	During the past year, have you been unable to remember what happened the night before because you had been drinking?	Never	0
			Monthly or less	1
			Monthly	2
			Weekly	3
			Daily or almost daily	4
9.	Have you injured yourself or anyone else because of your drinking?	Have you or someone else been injured as a result of your drinking?	No	0
			Yes, but not in the past year	2
			Yes, during the past year	4
10.	Has anyone (family, friend, doctor) been worried about your drinking or asked you to cut down?	Has a relative or friend, doctor or other health worker been concerned about your drinking or suggested you cut down?	No	0
			Yes, but not in the past year	2
			Yes, during the past year	4

Total AUDIT scores range from 0 to 40, with higher scores indicating more problematic alcohol use. A score of 8 or more was used to indicate at-risk drinking [5,11]. The Alcohol Treatment Guidelines for Indigenous Australians [5] uses a cutoff score of 13 or more to identify high-risk drinkers. This threshold is lower than the cutoff score of 16 or more suggested by WHO to identify a high level of problematic alcohol use [11]. It has been used widely in the Australian context to encourage earlier assessment for dependence, and because of the risk of social harms in Australians drinkers with AUDIT scores 12 and above [12]. However, to increase international comparability, and because of its likely closer reflection of the need for treatment of dependence, the higher WHO criterion was also applied (a score of 20 or more) for identifying a person as warranting further diagnostic evaluation for alcohol dependence. Therefore, participants were classified as either current nondrinkers (score = 0); low-risk drinkers (score = 1–7); at-risk drinkers (score = 8–12); high-risk drinkers (score = 13–19); or likely dependent drinkers (score ≥ 20).

AUDIT-C assesses frequency and quantity of alcohol use, and frequency of heavy drinking (six or more drinks on one day) (Table 1, items 1–3). Total scores range from 0 to 12. As with the 10-item AUDIT, higher scores indicate more problematic alcohol use. AUDIT-3 (the third item of the 10-item AUDIT) measures frequency of heavy drinking (Table 1, item 3). Total scores range from 0 to 4.

### Exclusion criterion

Participants who did not complete all 10 AUDIT items were excluded, except for those who appropriately did not answer item two because they indicated being a nondrinker on item one. In that circumstance, item two was scored 0, reflecting the participant’s status as a nondrinker.

### Data analysis

Sensitivity, specificity, and positive and negative predictive values [21] of each score on the AUDIT-C and AUDIT-3 were calculated, relative to cutoff scores on the 10-item AUDIT, as follows: at-risk drinkers (score ≥ 8); high-risk drinkers (score ≥ 13); and likely dependent drinkers (score ≥ 20) [5,8,11]. For this study, sensitivity is the proportion of respondents identified as problem drinkers on the 10-item AUDIT who are also identified as problem drinkers on AUDIT-C and/or AUDIT-3. Specificity is the proportion of respondents identified as nonproblem drinkers on the 10-item AUDIT who are also identified as nonproblem drinkers on AUDIT-C and/or AUDIT-3. The positive predictive value is the proportion of respondents identified as problem drinkers on AUDIT-C and/or AUDIT-3 who are also identified as problem drinkers on the 10-item AUDIT. The negative predictive value is the proportion of respondents identified as nonproblem drinkers on AUDIT-C and/or AUDIT-3 who are also identified as nonproblem drinkers on the 10-item AUDIT [21]. These analyses can identify false-positive and false-negative cases, meaning a false AUDIT-C or AUDIT-3 screen, relative to the 10-item AUDIT classification of problem drinker.

Receiver operating characteristic (ROC) curve analysis was conducted to measure the detection characteristics of AUDIT-C and AUDIT-3 for at-risk, high-risk, and likely dependent drinkers identified by the 10-item AUDIT [22]. A value of 1 for the Areas under the ROC (AUROC) curve represents a test with 100 percent accuracy. Ninety-five percent confidence intervals were calculated.

Data analysis was completed using IBM® SPSS® Statistics 19 [23], and Microsoft® Excel 2007 [24].

## Results

### Sample characteristics

One hundred and fifty-six Aboriginal Australian participants took part in the surveys, of whom 20 were excluded from the analyses: 16 because they did not answer all 10 items of AUDIT, and 4 were excluded in error (when the data were transferred from one computer program to another, 4 participants from the Sydney sample who had completed all 10 items of AUDIT but did not answer the question about gender were mistakenly excluded). Of the 136 participants in the final sample, 96 were recruited from rural NSW (37 from a drug and alcohol treatment agency, 58 from an ACCHS, and 1 did not indicate their recruitment source on the survey), and 40 were recruited from Sydney (all from existing Aboriginal community groups). Eleven percent of participants were age 18–24 years, 24 percent were age 25–34 years, 27 percent were age 35–44 years, 25 percent were age 45–55 years, 10 percent were 55 years or older (3% did not indicate their age), and 49 percent were male. Of the 20 participants excluded from the final sample (65% from rural NSW and 35% from Sydney), 10 percent were age 18–24 years, 25 percent were age 25–34 years, 20 percent were age 35–44 years, 5 percent were age 45–55 years, 5 percent were 55 years or older (35% did not indicate their age); and 45 percent were male (10% did not indicate their gender). There was a greater proportion of excluded participants who did not indicate their age and a smaller proportion of excluded participants who were age 45–55 years, compared to included participants.

### Alcohol use

AUDIT scores of participants ranged from 0 to 40 (median = 8.0; standard deviation = 11.0). Applying cutoff scores for the 10-item version of AUDIT resulted in the following distribution of participants across risk categories: 15 percent were nondrinkers (score = 0); 31 percent were low-risk drinkers (score = 1–7); 15 percent were at-risk drinkers (score = 8–12); 16 percent were high-risk drinkers (score = 13–19); and 22 percent were likely dependent drinkers (score ≥ 20). There were more at-risk drinkers in Sydney (30%) than in rural NSW (9%), and more likely dependent drinkers in rural NSW (30%), compared to Sydney (3%). Of the total sample, 73 (54%) were classified as being at least at-risk drinkers (AUDIT score ≥ 8), and 38 percent (n = 52) were classified as being at least high-risk drinkers (AUDIT score ≥ 13).

The distribution of participants across risk categories varied for males: 10 percent were nondrinkers; 18 percent were low-risk drinkers; 21 percent were at-risk drinkers; 16 percent were high-risk drinkers; and 34 percent were likely dependent drinkers. The distribution of participants also varied across risk categories for females: 21 percent were nondrinkers; 44 percent were low-risk drinkers; 10 percent were at-risk drinkers; 15 percent were high-risk drinkers; and 10 percent were likely dependent drinkers. The proportion of AUDIT-C score/AUDIT score ranged from 0.18 to 1 (mean = 0.56).

Excluded participants completed between 0 and 10 items of AUDIT, with an average of seven items completed (including the erroneously excluded participants who completed all 10 items). Eight of the 20 excluded participants completed the first three items of AUDIT. AUDIT-C total scores (first three items of AUDIT) for those eight participants ranged from 6 to 12 (mean = 7).

### At-risk drinkers

**Figure 1:**
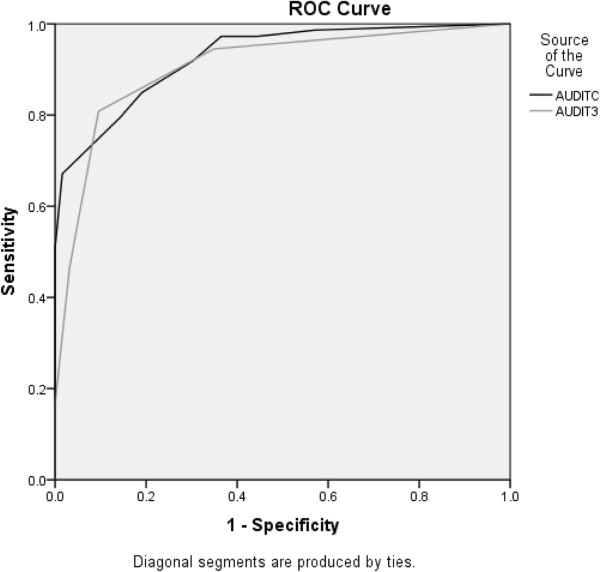
ROC curve for at-risk drinker (AUDIT score ≥ 8).

The AUROC for AUDIT-C was high for drinkers classified as being at increased risk by the 10-item AUDIT (0.93, 95% CI = 0.89 – 0.97) (Figure 1). The AUROC for AUDIT-3 also was high for drinkers classified as being at increased risk by the 10-item AUDIT (0.91, 95% CI = 0.85 – 0.96) (Figure 1). Table 2 shows the sensitivity, specificity, and positive and negative predictive values for at-risk drinkers and cutoff scores for AUDIT-C and AUDIT-3.

**Table 2:** **Measures of agreement for AUDIT-C and AUDIT-3 cutoff scores (n = 136**^**a**^**)**

	**Sensitivity**	**Specificity**	**Positive predictive value**	**Negative predictive value**	**Sensitivity**	**Specificity**	**Positive predictive value**	**Negative predictive value**	**Sensitivity**	**Specificity**	**Positive predictive value**	**Negative predictive value**
***Score (n)***	***At-risk drinker (10-item AUDIT score ≥ 8)***				***High-risk drinker (10-item AUDIT score ≥ 13)***				***Likely dependent drinker (10-item AUDIT score ≥ 20)***			
**AUDIT-C**												
≥ 1 (108)	0.99	0.43	0.67	0.96	0.98	0.32	0.47	0.96	1.00	0.26	0.28	1.00
≥ 2 (99)	0.97	0.56	0.72	0.95	0.98	0.43	0.52	0.97	1.00	0.35	0.30	1.00
≥ 3 (94)	0.97	0.63	0.76	0.95	0.98	0.49	0.54	0.98	1.00	0.40	0.32	1.00
≥ 4 (86)	0.92	0.70	0.78	0.88	0.96	0.57	0.58	0.96	1.00	0.47	0.35	1.00
≥ 5 (74)	*0.85*	*0.81*	*0.84*	*0.82*	0.92	0.69	0.65	0.94	0.97	0.58	0.39	0.98
≥ 6 (67)	0.79	0.86	0.87	0.78	*0.88*	*0.75*	*0.69*	*0.91*	0.97	0.64	0.43	0.99
≥ 7 (50)	0.67	0.98	0.98	0.72	0.81	0.90	0.84	0.88	0.93	0.79	0.56	0.98
≥ 8 (37)	0.51	1.00	1.00	0.64	0.63	0.95	0.89	0.81	0.87	0.90	0.70	0.96
≥ 9 (32)	0.44	1.00	1.00	0.61	0.58	0.98	0.94	0.79	*0.87*	*0.94*	*0.81*	*0.96*
≥ 10 (23)	0.32	1.00	1.00	0.56	0.42	0.99	0.96	0.73	0.63	0.96	0.83	0.90
≥ 11 (14)	0.19	1.00	1.00	0.52	0.27	1.00	1.00	0.69	0.47	1.00	1.00	0.87
≥ 12 (7)	0.10	1.00	1.00	0.49	0.13	1.00	1.00	0.65	0.23	1.00	1.00	0.82
**AUDIT-3**												
≥ 1 (91)	*0.95*	*0.65*	*0.76*	*0.91*	0.98	0.52	0.56	0.98	1.00	0.42	0.33	1.00
≥ 2 (65)	0.81	0.90	0.91	0.80	*0.92*	*0.80*	*0.74*	*0.94*	0.97	0.66	0.45	0.99
≥ 3 (36)	0.47	0.97	0.94	0.61	0.62	0.95	0.89	0.80	*0.93*	*0.92*	*0.78*	*0.98*
≥ 4 (12)	0.16	1.00	1.00	0.51	0.23	1.00	1.00	0.68	0.40	1.00	1.00	0.85

^a^156 Aboriginal Australian participants took part in the surveys, of whom 20 were excluded from the analyses: 16 because they did not answer all 10 items of AUDIT, and four were excluded in error (when the data were transferred from one computer program to another, four participants from the Sydney sample who had completed all 10 items of AUDIT but did not answer the question about gender were mistakenly excluded).

### High-risk drinkers

**Figure 2:**
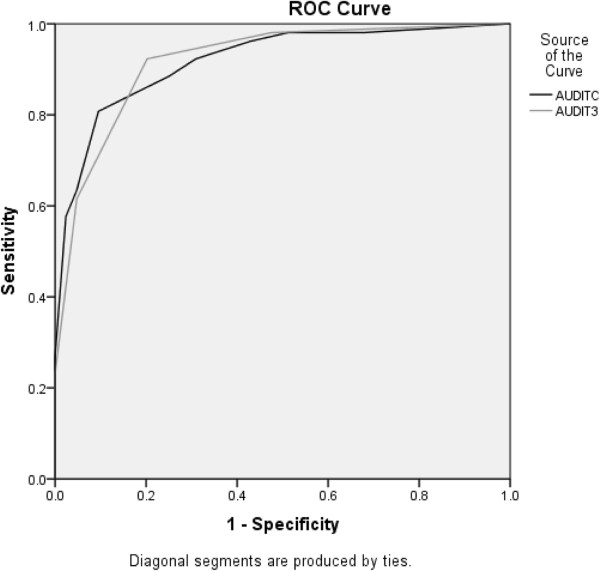
ROC curve for high-risk drinker (AUDIT score ≥ 13).

The AUROC for AUDIT-C was high for drinkers classified as being at high risk by the 10-item AUDIT (0.92, 95% CI = 0.87 – 0.97) (Figure 2). The AUROC for AUDIT-3 also was high for drinkers classified as being high risk by the 10-item AUDIT (0.92, 95% CI = 0.87 – 0.96) (Figure 2). Table 2 shows the sensitivity, specificity, and positive and negative predictive values for high-risk drinkers and cutoff scores for AUDIT-C and AUDIT-3.

### Likely dependent drinkers

**Figure 3:**
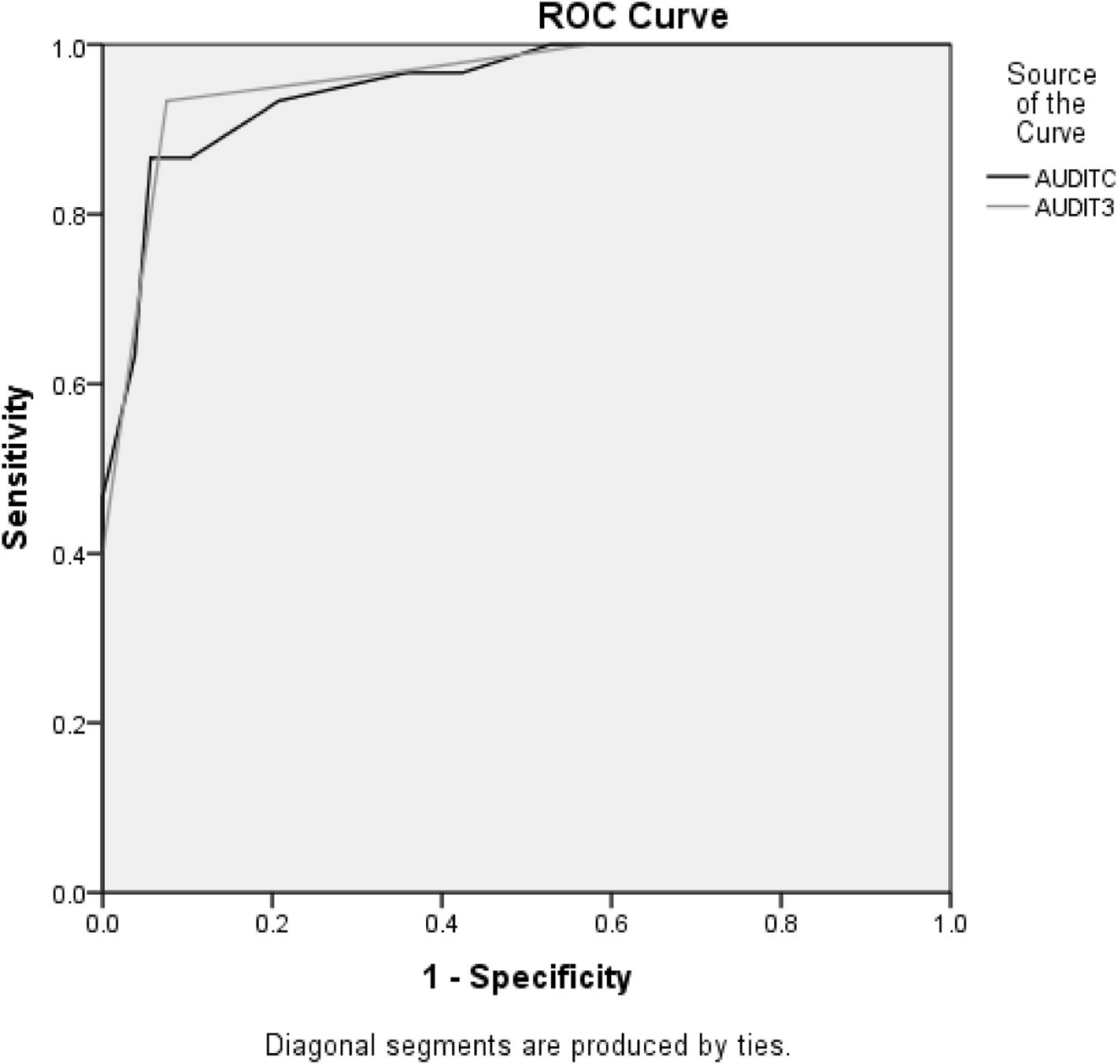
ROC curve for likely dependent drinker (AUDIT score ≥ 20).

The AUROC for AUDIT-C was high for respondents classified as being likely dependent drinkers by the 10-item AUDIT (0.95, 95% CI = 0.91 – 0.99) (Figure 3). The AUROC for AUDIT-3 also was high for respondents classified as being likely dependent drinkers by the 10-item AUDIT (0.96, 95% CI = 0.92 – 0.99) (Figure 3). Table 2 shows the sensitivity, specificity, and positive and negative predictive values for likely dependent drinkers and cutoff scores for AUDIT-C and AUDIT-3.

### Drink risk category classification

Table 3 presents the proportion of participants who were classified by AUDIT-C and AUDIT-3 into drink risk categories using cutoff scores suggested by this paper, compared to those classified into drink risk categories by the 10-item AUDIT.

**Table 3:** Drink status classified by the 10-item AUDIT, AUDIT-C, and AUDIT-3

**Drink status**	**Score**	**Total (n = 136**^**a**^**)**	**Male (n = 67)**	**Female (n = 68)**
**10-item AUDIT**				
Nondrinker	0	21 (15%)	7 (10%)	14 (21%)
Low-risk drinker	1–7	42 (31%)	12 (18%)	30 (44%)
At-risk drinker	8–12	21 (15%)	14 (21%)	7 (10%)
High-risk drinker	13–19	22 (16%)	11 (16%)	10 (15%)
Likely dependent drinker	≥ 20	30 (22%)	23 (34%)	7 (10%)
At least at-risk drinker	≥ 8	73 (54%)	48 (72%)	24 (35%)
At least high-risk drinker	≥ 13	52 (38%)	34 (50%)	17 (25%)
At least likely dependent drinker	≥ 20	30 (22%)	23 (34%)	7 (10%)
**AUDIT-C**				
At least at-risk drinker	≥ 5	74 (55%)	44 (66%)	29 (43%)
At least high-risk drinker	≥ 6	67 (49%)	40 (60%)	26 (38%)
At least likely dependent drinker	≥ 9	32 (24%)	22 (33%)	9 (13%)
**AUDIT-3**				
At least at-risk drinker	≥ 1	91 (67%)	52 (78%)	38 (56%)
At least high-risk drinker	≥ 2	65 (48%)	38 (57%)	26 (38%)
At least likely dependent drinker	≥ 3	36 (27%)	25 (37%)	1015%)

^a^156 Aboriginal Australian participants took part in the surveys, of whom 20 were excluded from the analyses: 16 because they did not answer all 10 items of AUDIT, and four were excluded in error (when the data were transferred from one computer program to another, four participants from the Sydney sample who had completed all 10 items of AUDIT but did not answer the question about gender were mistakenly excluded).

## Discussion

### Summary of results

This is the first study to identify the sensitivity, specificity, and positive and negative predictive values of AUDIT-C and AUDIT-3 for identifying problem drinkers, as determined by the 10-item AUDIT, among urban and rural Aboriginal Australians. The optimal combination of sensitivity and specificity for at-risk drinkers was reached using a cutoff score of ≥ 5 for AUDIT-C. This cutoff score identified 85 percent of at-risk drinkers, as classified by the 10-item AUDIT, and 81 percent of those identified as not being at increased risk. The positive and negative predictive values were both greater than 0.80. The optimal combination of sensitivity and specificity for at-risk drinkers was reached using a cutoff score of ≥ 1 for AUDIT-3. This cutoff score identified 95 percent of at-risk drinkers, as classified by the 10-item AUDIT, and 65 percent of those identified as not being at increased risk. A lower positive predictive value (0.76) than for AUDIT-C, however, indicated that a number of false-positive cases would be identified relative to the 10-item AUDIT. A cutoff score of ≥ 2 reduces the number of false-positive cases (positive predictive value = 0.91), but decreases the number of true-positive cases (sensitivity = 0.81) (Table 2). The optimal combination of sensitivity and specificity for high-risk drinkers was reached using a cutoff score of ≥ 6 for AUDIT-C. This cutoff score identified 88 percent of high-risk drinkers, as classified by the 10-item AUDIT, and 75 percent of those identified as not being at high risk. The positive predictive value (0.69) indicated a number of false-positive cases, relative to the 10-item AUDIT. If the cutoff score was increased to ≥ 7, the number of false-positive cases would be reduced (positive predictive value = 0.84), but the number of true-positive cases would be reduced (sensitivity = 0.88 using a cutoff score of ≥ 6 for AUDIT-C and 0.81 using a cutoff score of ≥ 7 for AUDIT-C). The optimal combination of sensitivity and specificity for high-risk drinkers was reached using a cutoff score of ≥ 2 for AUDIT-3. This cutoff score identified 92 percent of high-risk drinkers, as classified by the 10-item AUDIT, and 80 percent of those identified as not being at high-risk. The positive and negative predictive values were 0.74 and 0.94, respectively. The optimal combination of sensitivity and specificity for likely dependent drinkers was reached using a cutoff score of ≥ 9 for AUDIT-C. This cutoff score identified 87 percent of likely dependent drinkers, as classified by the 10-item AUDIT, and 94 percent of those identified as unlikely to be dependent drinkers. Positive and negative predictive values were both above 0.80. The optimal combination of sensitivity and specificity for likely dependent drinkers was reached using a cutoff score of ≥ 3 for AUDIT-3. This cutoff score identified 93 percent of likely dependent drinkers, as classified by the 10-item AUDIT, and 92 percent of those identified as unlikely to be dependent drinkers. Positive and negative predictive values were 0.78 and 0.98, respectively.

In summary, when using AUDIT-C to identify at-risk, high-risk, and likely dependent drinkers, as classified by the 10-item AUDIT, recommended cutoff scores are ≥ 5, ≥ 6, and ≥ 9, respectively. When using AUDIT-3 to identify at-risk, high-risk, and likely dependent drinkers, as classified by the 10-item AUDIT, recommended cutoff scores are ≥ 1, ≥ 2, and ≥ 3, respectively. All AUROCs were above 0.90, indicating good performance of both AUDIT-C and AUDIT-3 in identifying the at-risk, high-risk, and likely dependent drinkers.

Factors specific to Aboriginal health care settings were used to guide decisions about optimal cutoff scores. Since Aboriginal drinkers are more likely to drink at problematic levels than non-Aboriginal drinkers [2] there is a higher probability that Aboriginal drinkers will require an alcohol-specific intervention [5]. Consequently, higher sensitivity (to detect true-positive cases) took preference over higher specificity (to detect true-negative cases). For dependent drinkers, minimizing the number of people who receive referral unnecessarily is important, given their treatment is relatively expensive and typically involves multiple health care providers and inpatient care [5]. Consequently, higher specificity (to detect true-negative cases) was favored over higher sensitivity (to detect true-positive cases) for optimal cutoff scores in relation to likely alcohol dependence.

### Implications

Participants in this study were asked questions about number of drinks, rather than number of standard drinks, to cut down the need for mental arithmetic in a population that has often been educationally disadvantaged. The Australian standard drink is 10 g of ethanol, whereas a can of beer, for example, is approximately 1.3 standard drinks (13 g of ethanol), and most people drink wine in at least 1.8 standard drink servings (18 g of ethanol) [25]. It is unclear how the participants’ calculation of the number of drinks they consume compares to these standard drink definitions [26]. It is likely that participants under-reported their consumption as a result of the Aboriginal-friendly wording used for AUDIT; however, due to barriers to numeracy recognized within the Aboriginal Australian population, the increased ease of reporting drinks rather than calculating standard drinks is believed to outweigh the harms of under-reporting in Aboriginal health care settings [20].

The two results for which recommended cutoff scores are most difficult to determine are AUDIT-3 cutoff scores for at-risk drinkers and AUDIT-C cutoff scores for high-risk drinkers. The recommended cutoff scores prioritize higher sensitivity over higher specificity; however, this creates an issue of false-positives, which may result in additional work following up cases to distinguish false-positives from true-positives. If brief intervention is conducted appropriately and respectfully, however, then this follow-up process can be incorporated into discussions of current recommended drinking guidelines. A larger study may be able to more definitively determine appropriate cutoff scores in these cases.

From a clinical services perspective, a decision about which screening instrument is most appropriate for Aboriginal clients would be required, in consultation with Aboriginal health professionals and/or Aboriginal communities. For at-risk drinkers, AUDIT-C has a greater specificity, albeit a slightly lower sensitivity than AUDIT-3 (sensitivity: 0.85 and 0.95, respectively; specificity: 0.81 and 0.65, respectively), indicating a slight preference for using AUDIT-C to identify at-risk Aboriginal drinkers. For high-risk and likely dependent Aboriginal drinkers, using either AUDIT-C or AUDIT-3 would be appropriate, based on similar sensitivities and specificities for the two measures. The decision may be made on practical grounds: whether saving time during the screening process or in following up on positive results is more important. If screening is automated, with touch-screen computers for example, then the 3-item AUDIT-C (or indeed the 10-item AUDIT) may be desirable, given its greater specificity. If screening is manual, however, or screening is also required for a number of other health risk factors (e.g., smoking, nutrition, and obesity), asking only a single alcohol question (AUDIT-3) may be preferred, with a later discussion about drinking and other health risk factors during the clinical interview. Community consultation could help to determine which measurement tool is more acceptable to Aboriginal people in different circumstances.

### Limitations

A convenience sample was used. This method of recruitment, which resulted in a sample of Aboriginal Australians likely to access participating health services or community groups, probably resulted in low recruitment of treatment-resistant individuals with alcohol problems. Self-report data are prone to bias, even when this is minimized by using psychometrically validated tools [27]. Self-reported alcohol use is more likely to be accurate under optimal conditions: when participants are alcohol free; when they are assured confidentiality; when questions are clear; and in situations not likely to promote under-reporting (e.g., clinical compared to legal) [28]. These conditions were likely to be met by our study.

Although it has been recommended that measures to detect problematic alcohol use be tested separately for men and women [14], we did not complete gender-specific analyses due to the small number of men and women in each drink risk group. Given that lower cutoff scores have been recommended for women in other populations [14], these analyses would be worthwhile undertaking for studies with larger sample sizes.

The method used in this study of comparing results on AUDIT-C and AUDIT-3 to the 10-item AUDIT differs from other validation studies that compare the short versions of AUDIT with a ‘gold standard’ measure [14]. The method for this study, however, was required because a ‘gold standard’ measure for Aboriginal Australians is not available. The 10-item AUDIT questionnaire was used because it was recommended for use in Aboriginal health care settings [5], even though validated cutoff scores for Aboriginal Australians have not been published. This research used the AUDIT cutoff scores for at-risk and high-risk drinkers recommended by the Alcohol Treatment Guidelines for Indigenous Australians [5] and the cutoff score for likely dependent drinkers recommended by WHO [11]. The use of recommended AUDIT cutoff scores, in the absence of validated AUDIT scores, is a limitation of this research. The recommended cutoff scores for at-risk, high-risk, and likely dependent drinkers for AUDIT-C and AUDIT-3 explored in this paper should be used in parallel, rather than concurrently. In other words, the at-risk drinker cutoff score identifies drinkers who are at least at risk of alcohol-related harm (score ≥ 8); the high-risk drinker cutoff score identifies drinkers who are at least high-risk drinkers (score ≥ 13); and the likely dependent drinker cutoff score identifies drinkers who are likely to be dependent on alcohol (score ≥ 20). In a primary health care setting, one recommended cutoff score can be used to identify at-risk, high-risk, or likely dependent drinkers, depending on the need within that setting (rather than two or more recommended cutoff scores being used within that setting). These analyses investigating parallel cutoff scores provide an opportunity for the Aboriginal-specific recommended cutoff scores for at-risk and high-risk drinkers to be investigated, as well as the likely dependent drinker category recommended by WHO. Determining cutoff scores for AUDIT-C and AUDIT-3 that reflect drink risk categories of the 10-item AUDIT gives health care providers a better understanding of the strengths and limitations of AUDIT-C and AUDIT-3 for identifying problem drinkers in Aboriginal primary health care settings. There is error associated with the identification of problem drinkers using any of these screening instruments (AUDIT, AUDIT-C, or AUDIT-3), and individuals may potentially be allocated to different drink risk categories depending on which measure is used [29]. Therefore, healthcare providers should be aware that further alcohol questioning may be warranted if suggested by clinical experience. Comparison with other validation studies should be made with caution, because different results may have been found if AUDIT-C and AUDIT-3 were evaluated against a reference standard.

The AUDIT scoring was developed at a time when international and Australian drinking guidelines were more liberal. Further study is required to determine if the current recommended AUDIT cutoff scores (and hence AUDIT-C and AUDIT-3 cutoff scores) should be revised downward, to allow detection of anyone drinking over current recommended limits (e.g., 20 g daily or 40 g on any one occasion in Australia) [25], and whether the use of open-ended responses for questions one and two, which would provide noncategorized rather than categorized continuous measures of an individual’s quantity and frequency of alcohol consumption, results in a more accurate identification of their drink risk status. Given that Aboriginal people are unlikely to conceptualize and consume their alcohol as standard drinks [26], open-ended questions that establish what they drink, how much they drink, and their frequency of drinking are likely to be a more accurate measure than using categorized continuous measures.

Finally, the diagnostic error of the 10-item AUDIT questionnaire is expected to be highly correlated with the error of AUDIT-C and AUDIT-3 and, therefore, the AUROC analyses are likely to be biased upwards. Deriving AUDIT-C and AUDIT-3 scores from the 10-item AUDIT is also likely to have biased the AUROC analyses upwards. AUROCs in this study ranged from 0.91 to 0.96 and therefore, if the results were revised downwards to account for bias, they are still likely to be comparable to other validation studies [14].

## Conclusions

AUDIT-C and AUDIT-3 can be substituted for the 10-item AUDIT in Aboriginal health care settings using the following cutoff scores: at-risk drinkers, AUDIT-C ≥ 5 and AUDIT-3 ≥ 1; high-risk drinkers, AUDIT-C ≥ 6 and AUDIT-3 ≥ 2; and likely dependent drinkers, AUDIT-C ≥ 9 and AUDIT-3 ≥ 3. These findings provide a preliminary look into how brief screens compare with the 10-item AUDIT in best identifying varying levels of problematic alcohol use among Aboriginal Australians. There remains a need for studies that compare these brief screens with a ‘gold standard’ measure of problematic alcohol use using large, randomly selected and gender-stratified samples. However, before this can be achieved, a ‘gold standard’ measure of problematic alcohol use needs to be identified and validated within the Aboriginal Australian population.

## Abbreviations

ACCHS: Aboriginal Community Controlled Health Service; AUDIT: Alcohol Use Disorders Identification Test; AUROC: Area Under the Receiver operating characteristic; CI: Confidence interval; HREC: Human Research Ethics Committee; NSW: New South Wales; ROC: Receiver Operating Characteristic; WHO: World Health Organization.

## Competing interests

The authors declare that they have no competing interests.

## Authors’ contributions

BC was involved in developing the concept and design, data collection, and entry and analysis, and took the lead in drafting and revising the manuscript. AC was involved in developing the concept and design, data collection, and critically revising the manuscript. AS and KC were involved in developing the concept and design and critically revising the manuscript. LS, DB, and JA were involved in data collection and critically revising the manuscript. All authors approved the final version of the manuscript.
